# Transient Receptor Potential Vanilloid 4 Antagonist Eliminated Age-Related Spatial Memory Deficit in Female Sprague Dawley Rats

**DOI:** 10.1155/np/6405980

**Published:** 2025-11-25

**Authors:** Narongrit Thongon, Tanida Treerattanakulporn, Phossawee Kongkaew, Pongsakorn Lapchock, Nattida Kampuang, Siriporn Chamniansawat

**Affiliations:** Department of Medical Sciences, Faculty of Allied Health Sciences, Burapha University, No. 169 Long-Hard Bangsaen Rd., Saensook, Muang, Chonburi, Thailand

**Keywords:** aging, autophagy, inflammation, memory impairment, TRPV4

## Abstract

The expression of transient receptor potential vanilloid 4 (TRPV4) channels in the brains of normal-aging individuals significantly increases. However, the involvement of TRPV4 activity in age-related memory impairment remains unknown. This study aimed to investigate the role of TRPV4 in spatial memory tasks, hippocampal inflammation, and hippocampal autophagy in adolescent, adult, and aged female rats. Rats of different ages were used: 5, 10, 19, and 19 months treated with a TRPV4 inhibitor. Memory performance was assessed using the Morris water maze (MWM). Molecular changes were evaluated through western blotting, immunohistochemistry, and enzyme-linked immunosorbent assay (ELISA). The hippocampal TRPV4 expression was significantly increased in the aged rats. Furthermore, aged rats exhibited spatial memory decline, which was normalized with TRPV4 antagonist GSK2193874 (GSK219) injection. The senescence-associated β-galactosidase (SA-β-Gal) activity and hippocampal inflammatory cytokine and microglial activation marker levels in the hippocampus of aged rats were significantly increased. Similarly, the phosphoprotein marker levels of autophagy in the hippocampus of aged rats were significantly increased. GSK219 treatment effectively normalized hippocampal SA-β-Gal activity, inflammation, and autophagy in aged rats. TRPV4 hyperactivity was found to induce hippocampal inflammation and neuronal death, leading to spatial memory impairment in normal aging.

## 1. Introduction

Cognitive decline is a key feature of normal aging [1]. These include mitochondrial dysfunction, intracellular accumulation of oxidatively damaged proteins and nucleic acids, dysregulated energy metabolism, impaired cellular waste disposal mechanisms, impaired adaptive stress response signaling, compromised DNA repair, dysregulated neuronal Ca^2+^ homeostasis, stem cell exhaustion, and glia cell activation and inflammation [2]. Sustained neuronal inflammation and neuronal Ca^2+^ dysregulation were proposed as the main mechanisms underlying memory deficiency in normal aging [3–6]. Based on a previous study, age-related increases in inflammatory cytokines (e.g., interleukin [IL]-1, IL-6, IL-10, and tumor necrosis factor alpha [TNF-α]) were evident in the female hippocampus [7]. Nonsteroidal anti-inflammatory drugs significantly reduced neuroinflammation and improved memory in aged female humans [8] and aged mice [9]. In normal aging, neuronal Ca^2+^ dysregulation includes a higher Ca^2+^ influx and intracellular Ca^2+^ storage and a lower Ca^2+^ extrusion [4, 10, 11]. Verapamil, an L-type Ca^2+^ channel antagonist [12], and dantrolene, a ryanodine receptor antagonist [6], significantly improved spatial memory in aged mice.

Transient receptor potential vanilloid 4 (TRPV4), a nonselective cation channel with high Ca^2+^ permeability, is expressed in both neurons and glial cells throughout the brain and can be activated by diverse stimuli, including moderate heat (27–35°C), cell swelling, arachidonic acid metabolites, osmotic stress (hypo- and hyperosmolarity), and acidic pH (<6) [13, 14]. Through these properties, TRPV4 contributes to diverse physiological functions; however, its excessive activation promotes Ca^2+^ overload, leading to neurotoxicity and brain inflammation [15]. TRPV4 activation induces hippocampal neuronal apoptosis through a Ca^2+^-dependent mechanism [16]. In addition, TRPV4 signaling is closely linked to mTOR-regulated autophagy: under basal conditions, active mTOR phosphorylates and suppresses ULK1, thereby inhibiting autophagy [17]. TRPV4-mediated Ca^2+^ influx, however, promotes Ca^2+^-induced Ca^2+^ release from the endoplasmic reticulum [18], which activates the CaMKKβ/AMPK cascade, inhibits mTOR activity, and consequently initiates ULK1-dependent autophagy [17, 19, 20]. TRPV4 activation stimulates microglia and astrocytes, leading to the release of proinflammatory cytokines (IL-1β, IL-6, and TNF-α) and subsequent neuronal injury [21]. In a lipopolysaccharide-induced systemic inflammation model, TRPV4 hyperactivation promoted hippocampal inflammation and pyroptosis, resulting in cognitive impairment [22]. Conversely, pharmacological inhibition of TRPV4 suppressed glial activation, mitigated neuroinflammation and neuronal damage, and improved cognitive outcomes in models of systemic inflammation, demyelination, and seizures [22–25].

In normal aging, TRPV4 expression is upregulated in brain regions critical for memory processing, including the hippocampus [13]. Although this pattern implicates TRPV4 in age-related brain changes, its contribution to memory decline remains unclear. Dysregulated TRPV4 signaling disrupts synaptic integrity through Ca^2+^ overload, impaired mTOR–autophagy regulation, and inflammatory cascades. Supporting evidence from a sleep-deprivation model demonstrates that TRPV4 upregulation causes Ca^2+^ overload, reduces dendritic spine density, and downregulates PSD95, thereby impairing memory [22]. Collectively, these findings implicate TRPV4 dysregulation as a key driver of synaptic dysfunction and cognitive decline. Therefore, this study aimed to investigate how TRPV4 contributes to hippocampal plasticity, autophagy, and spatial memory impairment during normal aging.

Female Sprague Dawley rats were used to maintain consistency with our previous model [26, 27] and because females exhibit higher levels of proinflammatory cytokines, chemokines, and memory impairment compared to males [28]. To ensure adequate survival and avoid age-related comorbidities, we used rats aged below 20 months [29]. Experiments were conducted in 5-, 10-, and 19-month-old rats, corresponding to adolescence, adulthood, and aging, respectively [26, 30].

## 2. Materials and Methods

### 2.1. Animals

This study was performed in strict compliance with the Animal for Scientific Purposes Act of Thailand and in accordance with the Ethical Principles and Guidelines for the Use of Animals for Scientific Purposes, National Research Council of Thailand. The Ethics Committee on Animal Experiment of Burapha University, Thailand (ID#IACUC 029/2567), approved all experimental procedures. Female Sprague Dawley rats (aged 2 months old) were purchased from Nomura Siam International Co., Ltd., Thailand. The rats were acclimatized for 7 days and fed with standard pellet chow and reverse osmosis water ad libitum. The health, body weight, and food intake were monitored and recorded daily. The rats were randomly divided into four experimental groups: 5 months (5 MO), 10 months (10 MO), 19 months (19 MO), and 19 months plus the TPRV4 antagonist GSK2193874 (Sigma, St. Louis, MO, the United States; 19 MO + GSK219). The 19 MO + GSK219 group was injected daily with 1 mg/kg GSK219 intraperitoneally for 14 consecutive days [31, 32]. Meanwhile, the 5 MO, 10 MO, and 19 MO groups received daily intraperitoneal injection with the vehicle (2% dimethyl sulfoxide and 5% Tween 80; Sigma) [32] for 14 consecutive days. At 24 h prior to the experimental endpoint, the rats were housed in a metabolic cage to collect food and water intake. Further, information on urinary and fecal output was collected. The rats were anesthetized (70 mg/kg; Anesthal, Jagsonpal Pharmaceuticals Ltd., India), and blood sample was collected from the left ventricle. The rats were subsequently sacrificed. The hippocampus was harvested via rapid dissection and immediately frozen at 80°C for western blot analysis and enzyme-linked immunosorbent assay (ELISA).

### 2.2. Fasting Insulin and Glucose Levels

One week prior to the experimental end point, blood samples were collected from the saphenous vein under the 14-h fasting conditions. Samples were centrifuged at 700 g for 10 min and stored at 20°C until analysis. The fasting blood glucose level was measured using the Accu-Chek Performa glucometer (Roche Diagnostics, Mannheim, Germany). The fasting insulin concentration was determined using the ELISA kit (Thermo Fisher Scientific Inc.), according to the manufacturer's instructions.

### 2.3. Morris Water Maze (MWM)

The MWM, which is a robust and reliable test for hippocampal-dependent spatial memory, was performed using a method described in a previous study [26]. The rats were trained for 7 consecutive days. Thereafter, they were not disturbed for 7 days. On the testing day, the rats were allowed to swim in the pool for 60 s while the platform was removed.

### 2.4. ELISAs

ELISA was performed to measure IL-1β, IL-6, and TNF-α levels. The hippocampus was homogenized using an ultrasonic homogenizer in Pierce RIPA lysis buffer (Thermo Fisher Scientific Inc., Rockford, IL, USA) mixed with 1% protease inhibitor cocktail (Sigma). The samples were centrifuged at 12,000 × *g* for 15 min at 4°C, and the supernatants were collected. Total protein concentrations in the hippocampal supernatants were determined using the Bradford assay (Thermo Fisher Scientific Inc.) according to the manufacturer's instructions. The cytokine concentrations were normalized to total protein content (e.g., pg/mg protein). The IL-1β, IL-6, and TNF-α levels in the serum and supernatants were measured using commercial ELISA kits (Thermo Fisher Scientific Inc.), following the manufacturer's protocols.

### 2.5. Senescence-Associated β-Galactosidase (SA-β-Gal) Activity Assay

SA-β-Gal activity increases in senescent neurons and astrocytes [33]. The SA-β-Gal activity in the homogenized samples was measured using an assay kit (Ab287846; Abcam, Cambridge, the UK). The protocol for the standard curve and SA-β-Gal activity were followed-up according to the manufacturer's instructions. The hippocampus was homogenized using an ultrasonic homogenizer in ice-cold β-Gal Assay Buffer. The sample was centrifuged at 10,000 × *g* for 5 min at 4°C, and the supernatant was collected. A 10-µL sample was added into the 1:24 β-Gal substrate/β-Gal assay buffer mixture in a 96-well plate. The fluorescent signal was measured in the microplate reader.

### 2.6. Western Blot Analysis

Western blot analysis was performed using a method described in a previous study [26]. Hippocampal samples were lysed in cold Piece Ripa Buffer (Thermo Fisher Scientific Inc.) with 10% *v*/*v* protease inhibitor cocktail (Sigma) before sonication and centrifugation at 12,000 × *g* for 15 min. Proteins (30 mg) were separated by 10% sodium dodecyl sulfate polyacrylamide gel electrophoresis and were transferred to nitrocellulose membranes. The membrane was probed with 1:1000 primary antibodies raised against TRPV4 (Santa Cruz Biotechnology, Santa Cruz, CA, the USA), pSer2448-mTOR (Thermo Fisher Scientific Inc.), pSer317-ULK-1 (Thermo Fisher Scientific Inc.), pSer14-Beclin-1 (Abbiotec, San Diego, CA, the USA), Beclin-1 (Abbiotec), major histocompatibility complex II (MHC-II; Merck Millipore, Burlington, MA, the USA), cluster of differentiation molecule 11b (CD11b; Merck Millipore), CD45 (Merck Millipore), and ionized calcium-binding adapter molecule 1 (IBA-1; Merck Millipore), PSD-95 (MyBioSource, San Diego, CA, USA), and Bax (Abcam, Cambridge, UK). The membrane was subsequently incubated with 1:5000 horseradish peroxidase-conjugated secondary antibodies (Abcam, Cambridge, the UK), visualized with Thermo Scientific SuperSignal West Pico Substrate (Thermo Fisher Scientific Inc.), and captured using the ChemiDoc Touch Imaging System (Bio-Rad, Hercules, CA, the USA). Densitometric analysis was performed using ImageJ for Mac Os X [34].

### 2.7. Immunohistochemistry

Immunohistochemistry was performed using a method described in our previous study [26]. Rat hippocampal sections were incubated at 4°C overnight with 1:50 primary antibodies raised against TRPV4 (Santa Cruz Biotechnology). After washing, the sections were incubated for 1 h at room temperature with a biotinylated secondary antibody (Zymed, South San Francisco, California) at a 1:500 dilution. Then, they were incubated for 1 h with streptavidin-conjugated horseradish peroxidase solution (Zymed, South San Francisco, California, the USA) and 3,30-diaminobenzidine chromogen (Pierce, Rockford, Illinois). For negative controls, the sections were incubated with 0.7% Tween 20 in phosphate-buffered saline in the absence of the primary antibody. Finally, the sections were counterstained with hematoxylin (Sigma) and examined under a light microscope.

### 2.8. Statistical Analysis

Data were expressed as means ± standard errors of the mean. The two data sets were compared using the unpaired Student's *t*-test. One-way analysis of variance with Dunnett's posttest was used to compare multiple sets of data. All data were analyzed using GraphPad Prism (GraphPad Software Inc., San Diego, CA, USA).

## 3. Results

### 3.1. Metabolic Characteristics of the Rats

**Figure 1:**
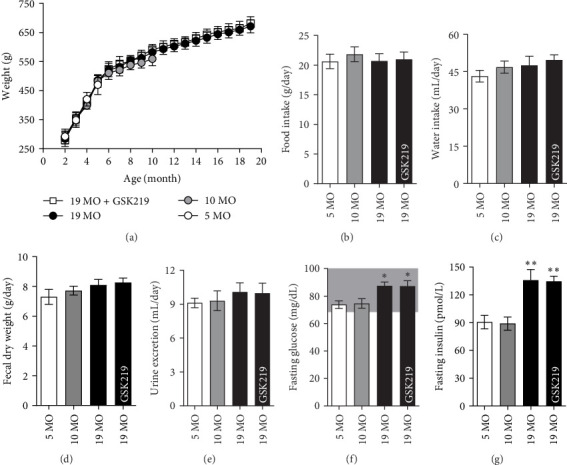
Metabolic characteristics. Body weight (a), food intake (b), water intake (c), fecal dry weight (d), urinary excretion (e), fasting blood glucose (f), and fasting blood insulin (g) in the 5 MO, 10 MO, 19 MO, and 19 MO + GSK219 groups. *⁣*^*∗*^*p* < 0.05, *⁣*^*∗∗*^*p* < 0.01 compared with the corresponding 5 MO group (*n* = 5).

During the experiment that lasted for 19 months, all rats were healthy, and their body weight increased (Figure 1a). The food intake (Figure 1b), water intake (Figure 1c), fecal excretion (Figure 1d), and urine excretion (Figure 1e) during the experiments did not differ. The fasting glucose (Figure 1f) and fasting insulin (Figure 1g) levels of the 19 MO and 19 MO + GSK219 groups significantly increased compared with those of the 5 MO group. All experimental groups had normal fasting glucose (70−99 mg/dL) and fasting insulin (<174 pmol/L) levels.

### 3.2. TRPV4 Antagonist Eliminated Age-Related Memory Impairment

**Figure 2:**
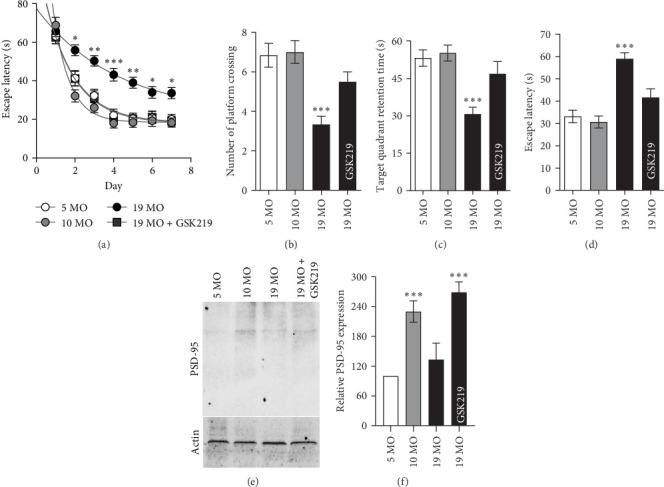
Spatial memory performance. Escape latency time over 7 days of training (a) in the 5 MO, 10 MO, 19 MO, and 19 MO + GSK219 groups. Number of platform crossings (b), target quadrant retention time (c), and escape latency time during the probe test (d). Representative western blot images of PSD-95 expression in the hippocampus (e) and quantitative analysis of relative PSD-95 expression levels (f) in the 5 MO, 10 MO, 19 MO, and 19 MO + GSK219 groups. *⁣*^*∗*^*p* < 0.05, *⁣*^*∗∗*^*p* < 0.01, and *⁣*^*∗∗∗*^*p* < 0.001 compared with the corresponding 5 MO group (*n* = 5).

Figure 2 presents the MWM study results obtained within 7 days of the training period (Figure 2a) and at the testing day, which was 7 days after the training period (Figure 2). Escape latency was defined as the time when animals find the platform and escape the maze, usually from Days 2–7 during training. The escape latency of the 19 MO group was significantly longer than that of the 5 and 10 MO groups (Figure 2a). The escape latency of the 19 MO + GSK219 group was comparable to that of the 5 MO group (Figure 2a). The number of platform crossings (Figure 2b) and target quadrant retention time (Figure 2c) of the 19 MO group were significantly lower than those of the 5 and 10 MO groups. The TRPV4 antagonist GSK219 significantly improved the number of platform crossings (Figure 2b) and the target quadrant retention time (Figure 2c) in the 19 MO group. The testing escape latency time of the 19 MO group was the highest and was restored by GSK219 (Figure 2d). Based on these results, the TRPV4 antagonist cured the spatial memory decrement in normal-aged female rats. In addition, PSD-95 expression, a critical postsynaptic density protein involved in dendritic spine stability and synaptic plasticity, was significantly reduced in the 19 MO group compared to the 5 MO and 10 MO groups (Figure 2e,f). Importantly, treatment with GSK219 markedly restored PSD-95 expression levels in aged rats, making them comparable to those of young controls.

### 3.3. Age-Related Hippocampal TRPV4 Expression

**Figure 3:**
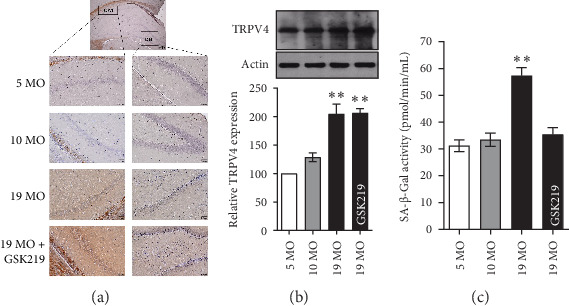
Hippocampal TRPV4 expression and SA-β-Gal activity. Immunohistochemistry (a) and western blot analysis (b) of TRPV4 expression in the hippocampus of the 5 MO, 10 MO, 19 MO, and 19 MO + GSK219 groups. Hippocampal SA-β-Gal activity (c) in the 5 MO, 10 MO, 19 MO, and 19 MO + GSK219 groups. *⁣*^*∗∗*^*p* < 0.01 compared with the corresponding 5 MO group (*n* = 5).

In the hippocampus of the 19 MO group, an increase in the immunoreactivity signal of TRPV4 was observed in the CA1 areas and dentate gyrus (Figure 3a). The immunoblotting analysis showed that the hippocampal TRPV4 expression of the 19 MO group significantly increased compared with that of the 5 MO group (Figure 3b). GSK219 did not affect age-related hippocampal TRPV4 expression in the 19 MO group (Figure 3a,b). The SA-β-Gal activity is not a fully specific marker of brain aging. However, it significantly increases the proportion of senescent neurons and astrocytes [33]. The SA-β-Gal activity of the 19 MO group significantly increased compared with that of the 5 MO group (Figure 3c). Surprisingly, the TRPV4 antagonist normalized the SA-β-Gal activity in the 19 MO group. Presumably, the inhibition of the TRPV4 activity could impede the senescence process in the hippocampus in normal aging.

**Figure 4:**
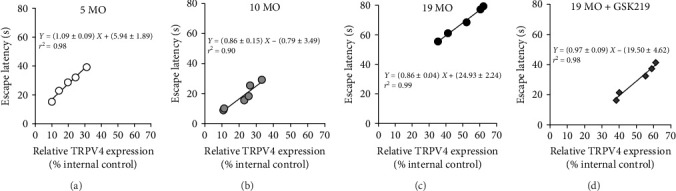
Relationship between hippocampal TRPV4 expression and spatial memory performance. Linear regression analysis of relative hippocampal TRPV4 expression and escape latency time during testing in the 5 MO (a), 10 MO (b), 19 MO (c), and 19 MO + GSK219 (d) groups.

This study also examined the association between the hippocampal TRPV4 expression and testing escape latency time in each rat via a linear regression analysis (Figure 4). The linear regression formulas were *Y* = (1.09 ± 0.09)*X* + (5.94 ± 1.89) (*r*^2^ = 0.98) in the 5 MO group; *Y* = (0.86 ± 0.15)*X* – (0.79 ± 3.49) (*r*^2^ = 0.90) in the 10 MO group; *Y* = (0.86 ± 0.04)*X* + (24.93 ± 2.24) (*r*^2^ = 0.99) in the 19 MO group; and *Y* = (0.97 ± 0.09)*X* – (19.50 ± 4.62) (*r*^2^ = 0.98) in the 19 MO + GSK219 group. Based on these results, the hippocampal TRPV4 expression was directly related to spatial memory. The TRPV4 expression was comparable. However, the TRPV4 antagonist also improved spatial memory in 19 MO rats. Therefore, there was an association between the spatial memory decrement and TRPV4 hyperactivity in normal-aged female rats.

### 3.4. TRPV4-Dependent Hippocampal Inflammation

**Figure 5:**
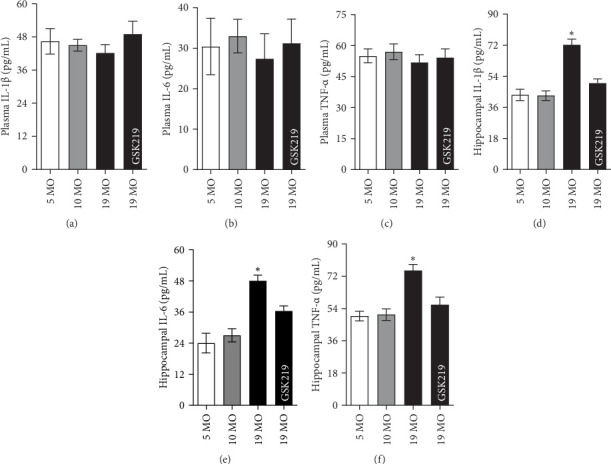
Hippocampal inflammation. Plasma IL-1β (a), plasma IL-6 (b), and plasma TNF-α (c) levels in the 5 MO, 10 MO, 19 MO, and 19 MO + GSK219 groups. Hippocampal IL-1β (d), hippocampal IL-6 (e), and hippocampal TNF-α (f) levels in the 5 MO, 10 MO, 19 MO, and 19 MO + GSK219 groups. *⁣*^*∗*^*p* < 0.05 compared with the corresponding 5 MO group (*n* = 5).

The role of the TRPV4 activity in systemic and hippocampal inflammation was examined. The plasma IL-1β (Figure 5a), plasma IL-6 (Figure 5b), and plasma TNF-α (Figure 5c) levels were comparable in all the experimental groups. Meanwhile, the expression of hippocampal IL-1β (Figure 5d), hippocampal IL-6 (Figure 5e), and hippocampal TNF-α (Figure 5f) in the 19 MO group significantly increased compared with that in the 5 MO group. GKS219 significantly suppressed the expression of hippocampal IL-1β (Figure 5d), hippocampal IL-6 (Figure 5e), and hippocampal TNF-α (Figure 5f) in the 19 MO group.

**Figure 6:**
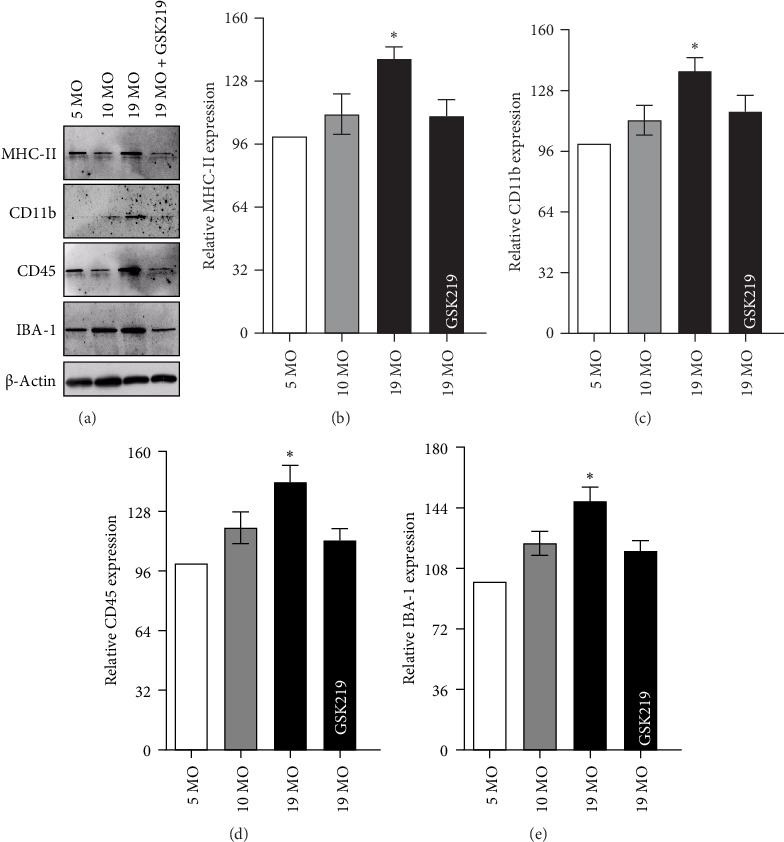
Hippocampal microglial activation. Western blot analysis and densitometric analysis of MHC-II (a, b), CD11b (a, c), CD45 (a, d), and IBA-1 (a, e) in the hippocampus of the 5 MO, 10 MO, 19 MO, and 19 MO + GSK219 groups. *⁣*^*∗*^*p* < 0.05 compared with the corresponding 5 MO group (*n* = 5).

Hippocampal microglia activation was determined by the expression levels of specific markers—e.g., MHC-II (Figure 6a,b), CD11b (Figure 6a,c), CD45 (Figure 6a,d), and IBA-1 (Figure 6a,e). The hippocampal MHC-II, CD11b, CD45, and IBA-1 expression in the 19 MO group significantly increased compared with that in the 5 MO group. Meanwhile, the TRPV4 antagonist significantly suppressed the expression of these microglia activation markers in the 19 MO group. Based on these results, the TRPV4 activity played a role in hippocampal microglia activation and inflammation in normal-aged female rats.

### 3.5. TRPV4-Dependent Hippocampal Autophagy and Bax Expression

**Figure 7:**
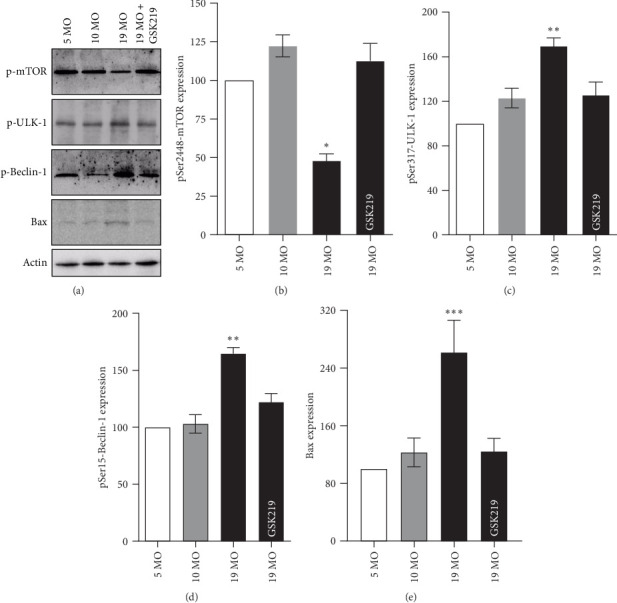
Hippocampal autophagy. Western blot analysis and densitometric quantification of phosphorylated proteins and apoptosis marker in the hippocampus of the 5 MO, 10 MO, 19 MO, and 19 MO + GSK219 groups. Representative blots show pSer2448-mTOR, pSer317-ULK-1, pSer15-Beclin-1, and Bax expression (a). Quantification of pSer2448-mTOR (b), pSer317-ULK-1 (c), pSer15-Beclin-1 (d), and Bax (e) expression across experimental groups. Data are presented as mean ± SEM. *⁣*^*∗*^*p* < 0.05, *⁣*^*∗∗*^*p* < 0.01, and *⁣*^*∗∗∗*^*p* < 0.001 compared with the corresponding 5 MO group (*n* = 5).

Active phospho-Ser2448-mTOR (pSer2448-mTOR) inhibits the autophagy pathway. Intracellular Ca^2+^ increments activated AMPK activity, which suppressed active pSer2448-mTOR and allowed AMPK-ULK-1 interaction. Subsequently, AMPK then phosphorylates ULK1 on the Ser317 residue (pSer317-ULK-1), which then activates ULK-1 kinase. This, in turn, activates Beclin-1 by increasing the expression of pSer14-Beclin-1 (pSer14-Beclin-1 in humans) and, eventually, leads to autophagy induction [17, 19, 20]. Therefore, the hippocampal expression levels of pSer2448-mTOR (Figure 7a,b), pSer317-ULK-1 (Figure 7a,c), pSer14-Beclin-1 (Figure 7a,d), and Bax (Figure 7a,e) were examined to elucidate TRPV4-related autophagy and apoptotic signaling. The hippocampal pSer2448-mTOR expression of the 19 MO group was significantly decreased compared with that of the 5 MO group, whereas pSer317-ULK-1 and pSer14-Beclin-1 expressions were significantly increased, indicating enhanced autophagy in aging. Importantly, Bax expression—a proapoptotic marker—was markedly elevated in the 19 MO group compared to the 5 MO and 10 MO groups, suggesting that TRPV4 hyperactivation contributes not only to autophagy induction but also to apoptosis in the aged hippocampus. Administration of the specific TRPV4 antagonist GSK219 restored pSer2448-mTOR expression and suppressed pSer317-ULK-1, pSer14-Beclin-1, and Bax expressions in the hippocampus of 19 MO rats. These findings indicate that TRPV4 hyperactivity drives hippocampal autophagy and proapoptotic signaling, contributing to hippocampal vulnerability during normal aging.

## 4. Discussion

The current study proposed that TRPV4 hyperactivation induced hippocampal inflammation and hippocampal autophagy, which led to age-related spatial memory decline in normal aged 19 MO female rats. The expression and activity of SA-β-Gal were significantly increased in the aging brain [34–36]. Based on a previous study, 18 and 24 MO rats had a higher SA-β-Gal expression in the hippocampus than 6 MO rats [35]. This finding is in accordance with the result of the current study, which showed that 19 MO female rats had a higher SA-β-Gal than 5 and 10 MO rats. The TRPV4 expression in the hippocampus of normal aged 24−29 MO male rats increased significantly [13], which was similar to our current results in normal aged 19 MO female rats. TRPV4 can be activated by diverse stimuli—e.g., moderate heat [27–35°C], arachidonic acid metabolites, and hypo- and hyperosmolar stimuli [13, 14]. The increase in TRPV4 stimuli (i.e., osmotic stress and arachidonic acid metabolites) has been reported in the aging brain [37, 38]. Thus, the hyperactivation and hyperactivity of TRPV4 should occur in the brain of normal-aging populations. Based on a recent report, the 19 MO groups had spatial memory impairment, which was recovered by the specific TRPV4 antagonist GSK219. Further, there was a direct association between spatial memory decline and the TRPV4 activity in normal-aged female rats. Based on these findings, TRPV4 hyperactivity played an essential role in age-related memory impairment in normal-aged female rats.

Although TRPV4 expression remained elevated after GSK219 administration, the functional recovery—such as improvement in spatial memory, reduction of hippocampal inflammation, and normalization of autophagy markers—strongly supports effective pharmacological blockade of TRPV4 activity. This pattern is consistent with previous studies, such as in the temporal lobe epilepsy model [39], where TRPV4 inhibition ameliorated pyroptosis-related outcomes without altering expression levels. Therefore, changes in pathological readouts serve as valid indirect evidence for successful TRPV4 inhibition in vivo. Based on these findings, TRPV4 hyperactivity played an essential role in age-related memory impairment in normal-aged female rats.

Importantly, the current study highlights a potential link between TRPV4 hyperactivation and age-related deficits in neural plasticity. Although TRPV4 has been implicated in CNS pathology, its explicit role during the aging process remains poorly understood. Our findings demonstrate that TRPV4 hyperactivity in the aged hippocampus is associated with dysregulation of autophagy, increased neuroinflammation, and reduced spatial memory performance, all of which are known contributors to impaired synaptic plasticity. Consistent with these findings, we observed a significant reduction of PSD-95 expression in aged rats, indicating synaptic destabilization and impaired postsynaptic scaffolding. Treatment with GSK219 restored PSD-95 levels to near-young control values, suggesting that TRPV4 blockade helps preserve synaptic structure and plasticity. This suggests that excessive TRPV4 activity may negatively influence hippocampal network remodeling and long-term potentiation (LTP), thereby accelerating age-associated cognitive decline. Furthermore, the reversal of these phenotypes by GSK219 supports the hypothesis that pharmacological inhibition of TRPV4 preserves hippocampal integrity and maintains plasticity-related mechanisms in normal aging. Future investigations integrating electrophysiological and calcium-imaging approaches could further clarify how TRPV4 modulates synaptic efficacy and structural remodeling during the aging process.

Neuroinflammation is triggered by the activation of microglia and astrocytes [40, 41], both of which secrete inflammatory cytokines (e.g., IL-1β, IL-6, and TNF-) into the brain extracellular fluid. The specific TRPV4 agonist GSK1016790A triggered hippocampal microglia and astrocyte activation, which, in turn, induced hippocampal inflammation [21]. Our results showed that the hippocampal inflammatory cytokines (IL-1β, IL-6, and TNF-α) and microglia activation markers (MHC-II, CD11b, CD45, and IBA-1) remarkably increased in normal-aged 19 MO female rats. The injection of specific TRPV4 antagonist GSK219 suppressed the expression of both inflammatory cytokines and microglia activation markers in the hippocampus of normal-aged 19 MO female rats. A recent study proposed that TRPV4 hyperactivation induced hippocampal inflammation in normal-aged female rats.

Previous studies have revealed that the active TRPV4 channel induced autophagy in rat hepatic stellate cells, human glioma cells, and osteoclastic cells [39, 42, 43]. The current study reported that TRPV4 hyperactivity induced hippocampal autophagy in normal-aged female rats. TRPV4 hyperactivation induced hippocampal inflammation and hippocampal proptosis [37]. Therefore, TRPV4 hyperactivation caused hippocampal neuronal death, which led to cognitive impairment in aging [37]. In conclusion, TRPV4 hyperactivity induced hippocampal inflammation and hippocampal neuronal death that led to spatial memory impairment in normal aging. The inhibition of the TRPV4 activity improved spatial memory in normal aging.

A limitation of the current study is the absence of direct measurements of TRPV4 channel activity in the hippocampus. However, the use of a highly selective antagonist (GSK219), combined with the reversal of TRPV4-related phenotypes, provides strong inferential evidence supporting functional inhibition. Another limitation is that systemic administration of GSK219 does not allow us to conclusively distinguish between central and peripheral TRPV4 blockade. Although our findings strongly support hippocampal TRPV4 hyperactivation as a driver of neuroinflammation, autophagy dysregulation, and synaptic impairment, the possibility that peripheral TRPV4 inhibition also contributes to the observed behavioral and molecular effects cannot be excluded. Future studies employing electrophysiological recordings, calcium-imaging techniques, region-specific drug delivery, or TRPV4 conditional knockout models will be essential to validate these findings and to delineate the relative contributions of central versus peripheral mechanisms.

## Data Availability

All data generated or analyzed during this study are included in this published article. Further inquiries data can be directed to the corresponding author.
